# Low mir-372 expression correlates with poor prognosis and tumor metastasis in hepatocellular carcinoma

**DOI:** 10.1186/s12885-015-1214-0

**Published:** 2015-03-26

**Authors:** Gang Wu, Yawei Wang, Xiaojun Lu, Hui He, Haiyang Liu, Xiangyu Meng, Shuguan Xia, Kunming Zheng, Boqian Liu

**Affiliations:** 1 Department of General Surgery, The First Affiliated Hospital of China Medical University, Shenyang, Liaoning 110001 China; 2 Department of General Surgery, Changhai Hospital Affiliated to Second Military Medical University, Shanghai, China; 3 Department of General Surgery, The First Hospital Affiliated to Dalian Medical University, Dalian, China; 4 Department of General Surgery, The Fourth Hospital Affiliated to China Medical University, Shenyang, Liaoning China

**Keywords:** microRNA-372, HCC, Prognosis, Proliferation, Invasion

## Abstract

**Background:**

Recent studies have shown that miR-372 plays important roles in hepatocellular carcinoma (HCC) progression. However, results have been conflicting regarding its expression levels and role in HCC.

**Methods:**

RT-PCR and *in situ* hybridization was used to evaluate miR-372 expression in HCC tissues and cell lines. The methylation status of neighboring CpG islands upstream of the miR-372 promoter was analyzed by methylation-specific PCR (MSP). Transfection of miR-372 mimic into HCC cell lines was used to evaluate cellular proliferation and invasion. Prognostic significance was analyzed by the Kaplan–Meier survival method and Cox regression.

**Results:**

miR-372 was expressed at lower levels in HCC tissues compared with controls and was related to tumor metastasis and poor prognosis. Hypermethylation of miR-372 was detected in HCC cell lines and tissues, and miR-372 expression was restored upon 5-aza-dCyd treatment. Upregulated expression by mir-372 mimic transfection inhibited proliferation and invasion capacity in HCC cells.

**Conclusions:**

miR-372 may play an important role in hepatic carcinogenesis and may serve as a new target or method to detect and treat HCC in the future.

## Background

Hepatocellular carcinoma (HCC) is the fifth most common malignant tumor worldwide with an incidence of approximately 626,000 cases each year [1,2]. In China and Southeast Asia, HCC is highly associated with viral hepatitis B and cirrhosis [3]. Prognosis of patients with HCC has improved largely owing to the development of effective surgical techniques and diagnostic methods over recent years. However, long-term prognosis is still unsatisfactory largely due to the high recurrence and invasion rates even after resection (50–70% at 5 years) [4,5].

MicroRNAs (miRNAs) represent a class of endogenous, highly conserved, small nonprotein-coding RNAs that are approximately 22 nucleotides in length [6]. miRNAs are expressed in many organisms and function in the regulation of target gene expression via a complex process [7,8]. miR-372 belongs to the Mir-371-372 gene cluster, which is located on chromosome 19q13.42 [9]. Recent studies demonstrated that miR-372 regulates the cell cycle, apoptosis, invasion, and proliferation in many types of human cancers. For example, miR-372 promotes cell proliferation and cell cycle progression, and suppresses apoptosis in testicular germ cell tumors as well as in a gastric cancer cell line [10]. Yu et al. [11] identified miR-372 as a prognostic marker for the prediction of cancer relapse and survival in non-small cell lung cancer patients independent of stage or histological type. Furthermore, Lai et al. [12] provided evidence that miR-372 may post-transcriptionally downregulate the large tumor suppressor homolog 2 in non-small cell lung cancer patients resulting in tumorigenesis and proliferation. However, the role of miR-372 in HCC has not been clear. Gu [13] reported that miR-372 was expressed at high levels in HCC and may exert a proto-oncogene effect in hepatic carcinogenesis. In contrast, our previous study showed opposite results, and demonstrated that mir-372 was expressed in HCC at low levels and plays an anti-oncogene role by negatively controlling its target gene ATAD2 [14].

In this study, we used various methods to evaluate miR-372 expression levels, as well as investigate the mechanism of its abnormal expression and role in HCC.

## Methods

### Patient tissue samples and liver cancer cell lines

HCC tissue slice samples were obtained from 120 patients (51 males and 69 females) diagnosed with HCC who had undergone a routine hepatic resection in the First Affiliated Hospital of China Medical University between January 2007 and January 2009. The follow-up period for survivors was 5 years. None of the patients had received preoperative radiotherapy or chemotherapy prior to surgical resection. A total of 37 paired fresh specimens, including both tumor tissues and the corresponding paired noncancerous parenchyma, were snap-frozen in liquid nitrogen and stored at −70°C immediately after resection until processing. Histological diagnosis and differentiation were evaluated independently by three pathologists according to the WHO classification system [15]. The project protocol was approved by the Institutional Ethics Committee of China Medical University prior to the initiation of the study. All patients provided written informed consent for the use of the tumor tissues for clinical research. The liver cancer cell lines Huh7, HCCLM3, HepG2, SMMC7721, PLC5, and QGY7701 and the normal liver cell line LO2 were obtained from Shanghai Cell Bank (Shanghai, China).

### RNA preparation and quantitative real-time PCR

Total RNA was extracted from approximately 100 mg of the 37 paired tissue samples and liver cancer cell lines using TRIzol reagent ( Invitrogen Company,USA) according to the manufacturer’s instructions. The miR-372 primers were purchased from Takara Company (Japan). The U6B gene was used as a reference control for miR-372. The relative levels of gene expression were represented as ΔCt = Ct gene - Ct reference and the fold change in gene expression was calculated using the 2^−ΔΔCt^ method [16].

### DNA extraction and methylation-specific PCR (MSP)

Genomic DNA was extracted from cell lines and specimens using SDS/proteinase K, followed by phenol-chloroform extraction and ethanol precipitation. Bisulfite treatment was performed using the EZ DNA Methylation-Gold Kit (Zymo Research) according to the manufacturer’s instructions. The LO2 normal liver cell line and peripheral blood treatment with M. Sss I (New England Biolabs, Ipswich, USA) were used as negative and positive controls, respectively. A water control was run with every MSP. The analyst performed the procedures on 3 days.

### *In situ* hybridization

The *in situ* hybridization kit was purchased from Boster Company (Wuhan, China) and used according to the manufacturer’s instructions. Briefly, the tissue slides were hybridized with 20 ul of 5′-digoxigenin (DIG) LNA-modified-miR-372-3p. The nucleic acid sequence is 5′-ACGCTCAAATGTCGCAGCACTTT-3′. Results were independently scored by two experienced pathologists. The scoring of positive tumor cells was as follows: 0 (0%), 1 (1–10%), 2 (11–50%) and 3 (>50%). The staining intensity was visually scored as follows: 0 (negative), 1 (weak), 2 (moderate) and 3 (strong). The miR-372 expression score was calculated from the value of percent positivity score multiplied by the staining intensity score. This value ranged from 0 to 12, and the tumors were classified as follows: negative (−), score 0; lower expression (1+), score 1–4; moderate expression (2+), score 5–8; and strong expression (3+), score 9–12. *In situ* hybridization miR-372 staining was grouped into two categories: low expression (0/1+) and high expression (2+/3+).

### Cell transfection

For RNA transfection, 5 × 10^4^ HUH7 or HCCLM3 cells were seeded into each well of culture plates and incubated overnight. When cells were grown to 60–80% confluence, miR-372 mimic or negative control oligonucleotides (Genepharma Company) (5 pmol/μl) were transfected using Lipofectamine® RNAiMAX Reagent (Invitrogen, USA) according to the manufacturer’s instructions. Sequences are as follows: miR-372 mimic 5′-AAAGUGCUGCGACAUUUGAGCGUGCUCAAAUGUCGCAGCACUUUUU-3′, and miR-372 inhibitor 5′-ACGCUCAAAUGUCGCAGCACUUU-3′ (both purchased from Shanghai Genepharma Company). Cells plated in 96-well, 24-well, and 6-well plates were transfected with 1 μl (5 pmol), 3 μl (15 pmol), and 15 μl (75 pmol) oligonucleotides, respectively.

### Cell cycle analysis

Huh7 or HCCLM3 cells seeded at a density of 5 × 10^5^ per well in 6-well plates were transfected with miR-372 mimic or negative control. After 48 h of transfection, cells were trypsinized, fixed with 70% ethanol at 4°C, and washed with PBS. RNase A (100 μL) was added, and the mixture was incubated in a 37°C water bath for 30 min. Next, 400 μL PI staining solution was added and samples were incubated at 4°C in the dark for 30 min; a computer was then used to detect and record the red fluorescence upon excitation at a wavelength of 488 nm.

### CCK8 and colony formation assay

Cells were plated in 96-well plates in media containing 10% FBS at approximately 2,000 cells per well, 24 h after transfection. Next, 10 μl of CCK8 (thiazolyl blue) solution was added to each well and samples were incubated for 1 h at 37°C. The results were quantified spectrophotometrically using a test wavelength of 450 nm. After transfection, logarithmic growth phase cells in monolayer culture were prepared for the colony formation assay. Cells were plated in 6-well plates in media containing 10% FBS at approximately 200 cells per well. Colony formation was then allowed to proceed for 2 w. Cells were washed with 1 ml of PBS, fixed, stained with 500 μl of 0.1% crystal violet solution for 20 min, and finally washed three times with 1 ml of water. The fixed cell colonies were allowed to air dry. The clone formation rate was calculated.

### Cell invasion assay

Huh7 or HCCLM3 cells were infected with miR-372 mimic for 48 h. Cells were then seeded onto a synthetic basement membrane in the inset of a 24-well culture plate. In the invasion assay, polycarbonate filters coated with 50 μL Matrigel (1:9, BD Bioscience) were placed in a Transwell chamber (Costar). After incubation, filters were fixed and stained with 0.1% crystal violet solution. Non-invading cells were removed using a cotton swab, and invading cells on the underside of the filter were counted with an inverted microscope.

### Ethics statement

The project protocol was approved by the Institutional Ethics Committee of China Medical University prior to the initiation of the study. All patients provided written informed consent for the use of the tumor tissues for clinical research.

## Results

### miR-372 expression in HCC tissues and cell lines

**Figure 1:**
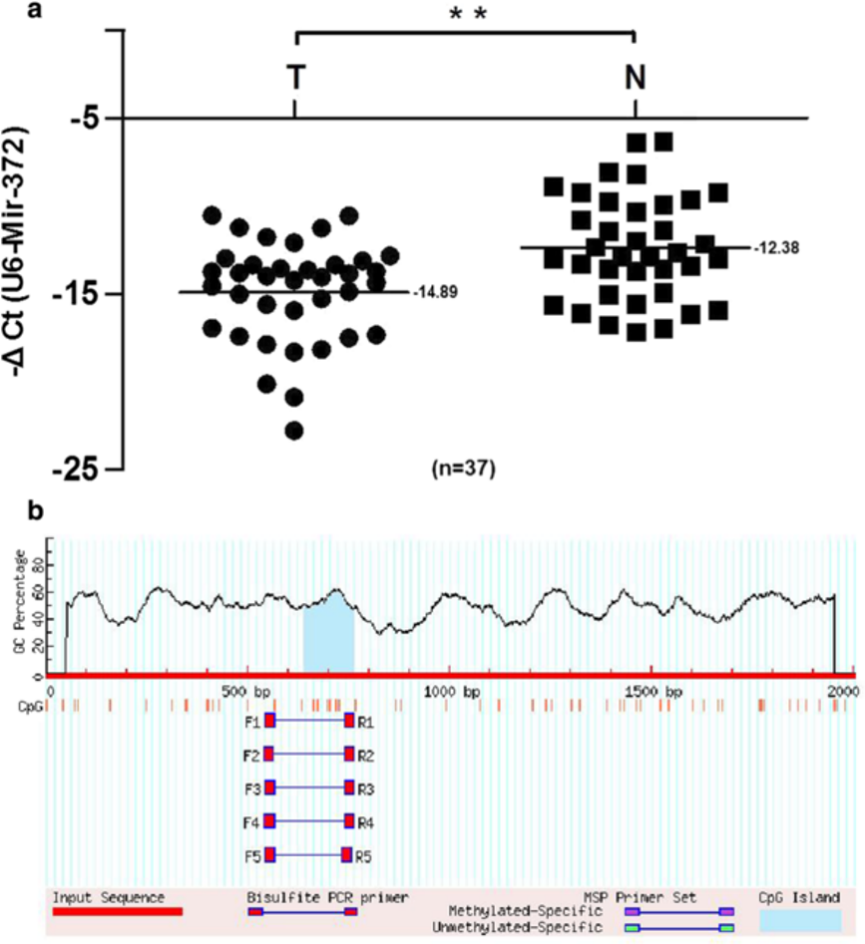
RT-PCR tested Mir-372 expression in 37 HCC tissues samples and corresponding normal tissues (−14.89 ± 2.83 vs −12.38 ± 2.96, **P < 0.01). a: ΔCt (U6B minus mir-372) was used to compared the expression difference between tumor and normal tissues (−14.89 ± 2.83 vs −12.38 ± 2.96, **P < 0.01); **b:** CpG islands located approximately 1,200 bp upstream of the promoter.

**Figure 2:**
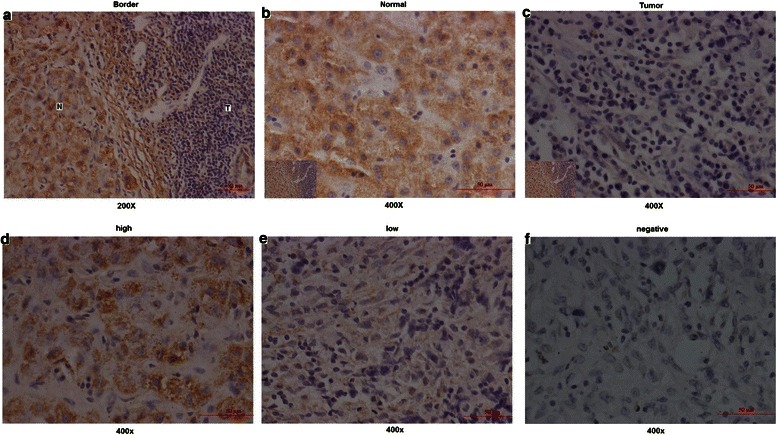
**In situ hybridization tested Mir-372 expression in 120 HCC tissue slice samples. a,b,c:** showed the staining difference between tumor and normal tissue slices; **d,e,f**: showed the different Mir-372 expression levels in tumor tissue slices.

**Figure 3:**
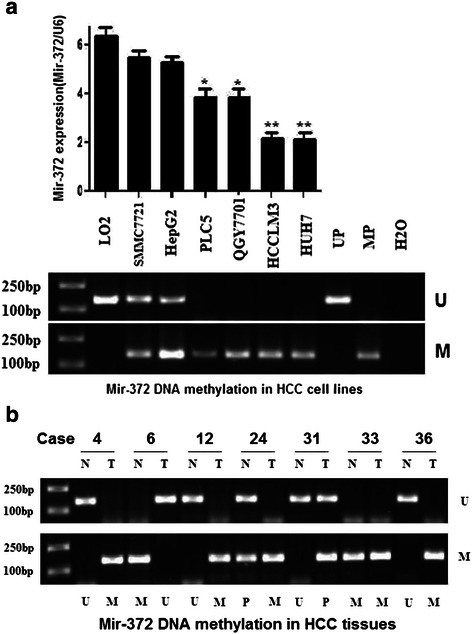
**RT-PCR and MSP respectively detected Mir-372 expression and DNA methylation. a:**Mir-372 expression levels and DNA methylation status in HCC cell lines. **b;** Mir-372 DNA methylation levels in HCC tumor and corresponding normal tissues. (U: unmethylation; M: methylation; N: corresponding normal tissues; T: tumor tissues; UP: negative controls; MP: positive controls).

RT-PCR showed that the mean expression levels of miR-372 were lower in HCC tissues compared with normal tissues (−14.89 ± 2.83 vs. −12.38 ± 2.96, respectively; *P* <0.01) (Figure 1). Of 120 HCC patients, miR-372 was expressed at low levels in 87 cases (72.5%) according to *in situ* hybridization (Figure 2). Furthermore, HUH7 and HCCLM3 HCC cell lines showed lower expression levels of miR-372 than HepG2, SMMC7721, PLC5, QGY7701 and LO2 cells (Figure 3a).

### Low expression of mir-372 is related to DNA methylation

To evaluate the mechanism of low expression of miR-372, we analyzed the upstream sequence of the miR-372 gene promoter and found CpG islands located approximately 1,200 bp upstream of the promoter. Thus we speculated that epigenetic changes of the miR-372 gene by aberrant promoter hypermethylation might be responsible for miR-372 low expression. We next analyzed the methylation status of miR-372 using MSP in both HCC tissue samples and cell lines. MSP analysis results showed hypermethylation of miR-372 in HUH7, HCCLM3, QGY7701, and PLC5 cells and partial methylation in Smmc7721 and HepG2 cells. However, no methylation was observed in LO2 cells. The methylation status of mir-372 in HCC cell lines was related to the expression level (Figure 3a).

**Figure 4:**
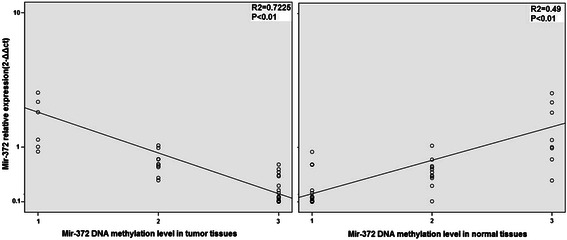
**The relationship between relative mir-372 expression in 37 cases HCC patients and DNA methylation level in tumor tissues and corresponding normal tissues.** It could be observed the DNA methylation level in tumor tissues was positive correlation with relative mir-372 expression, and DNA methylation level in normal tissues was negative correlation.

Among the 37 HCC specimens, 20 cases (54.1%) showed DNA hypermethylation of miR-372, and partial methylation was observed in 10 cases (27.0%) cases and 7 cases (18.9%) showed no methylation. In corresponding nonmalignant liver tissues, 9 cases (24.3%) showed hypermethylation, and partial methylation was observed in 11 cases (29.7%) cases and 17 cases (45.9%) showed no methylation. The DNA methylation level in tumor tissues showed a positive correlation with the relative miR-372 expression in 37 HCC cases and a negative correlation in normal tissues. This indicated that DNA methylation level not only affected miR-372 expression in tumor or normal tissues, but also the relative miR-372 expression difference between tumor and the corresponding normal tissues (Figure 4).

**Figure 5:**
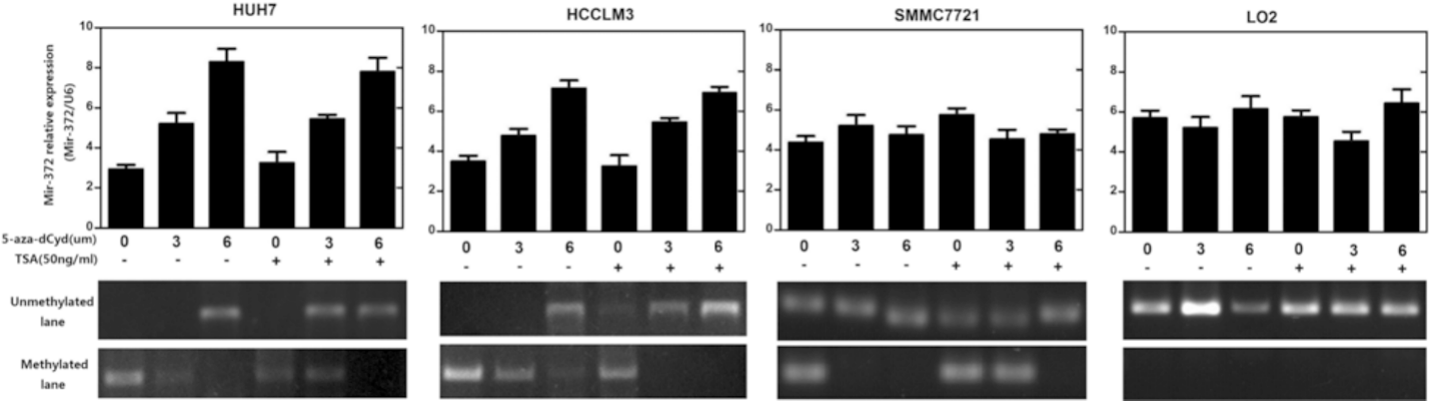
**Four HCC cell lines (HUH7, HCCLM3, SMMC7721, LO2) were treated with 5-aza-dCyd, a methyltransferase inhibitor and the miRNA expression levels were assayed using TaqMan miRNA PCR.** The expression of mir-372 was restored with 5-aza-dCyd treatment in HUH7 and HCCLM3 cell lines (*P < 0.05,**P < 0.01) and the mir-372 DNA methylation level was inhibited. Treatment with a histone deacetylase inhibitor, TSA, had no influence on the expression of mir-372 in all four cell lines.

We then assessed the effects of demethylation on the expression of miR-372. Four HCC cell lines (HUH7, HCCLM3, SMMC7721, LO2) were treated with 5-aza-dCyd, a methyltransferase inhibitor, and the miRNA expression levels were assayed using TaqMan miRNA PCR. The expression of miR-372 was restored with 5-aza-dCyd treatment in HUH7 and HCCLM3 cell lines and the miR-372 DNA methylation level was inhibited (Figure 5), suggesting that aberrant DNA methylation suppressed the expression of mir-372. Treatment with a histone deacetylase inhibitor, TSA, had no influence on the expression of miR-372 in all four cell lines (Figure 5). These findings suggest that histone deacetylation may not contribute to the transcriptional repression of miR-372.

### miR-372 expression was related to tumor metastasis and poor prognosis in HCC

**Figure 6:**
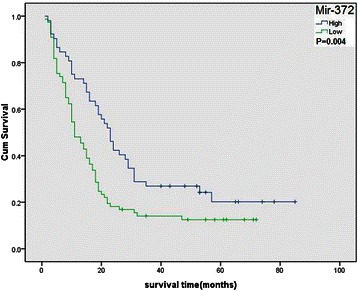
**Overall survival of HCC patients in relation to mir-372 expression levels according to in situ hybridization.** Survival of HCC patients with high mir-372 expression versus low expression.

We next analyzed the association between the miR-372 expression and various clinicopathological factors of the HCC patients. Interestingly, the expression levels of miR-372 were significantly lower in HCC tissues with advanced TNM stage compared with those with early TNM stage (*P* = 0.003) and miR-372 low expression tissues had more tumor metastasis (*P* < 0.001, Table 1). Patients with lower miR-372 expression had poorer prognosis. The overall survival was significantly lower in patients with low miR-372 expression than in patients with high miR-372 expression (*P* = 0.004) (Figure 6). In addition, multivariate analysis demonstrated that miR-372 levels, metastasis and AFP status were significant prognostic factors for HCC patients (Table 2).

**Table 1:** **Association between miR-372 expression according to in situ hybridization and conventional clinicopathological parameters in 120 patients with HCC**

Characteristics	Number of patients	Mir-372 Lowexpression	Mir-372 Highexpression	P
Total cases	120	87	33	
Age (years)				
≥50	74	54(72.9%)	20(27.1%)	0.883
<50	46	33(71.7%)	13(28.3%)	
Gender				
Male	51	37(72.5%)	14(27.5%)	0.992
Female	69	50(72.4%)	19(27.6%)	
Tumor size				
≥5 cm	51	33(64.7%)	18(35.3%)	0.100
<5 cm	69	54(78.3%)	15(21.7%)	
Metastasis				
Yes	37	32(86.5%)	5(13.5%)	**0.022**
No	83	55(66.3%)	28(33.7%)	
HBsAg status				
Positive	72	54(75%)	18(25%)	0.453
Negative	48	33(68.8%)	15(31.3%)	
Tumor differentiation				
High	43	29 (67.4%)	14(32.6%)	0.411
Moderate	44	35(79.5%)	9(20.5%)	
Poor	33	23(69.7%)	10(30.3%)	
Cirrhosis				
Yes	73	49(67.1%)	24(32.9%)	0.101
No	47	38(80.9%)	9(19.1%)	
Serum AFP				
<200 ng/dl	68	53(77.9%)	15(22.1%)	0.127
≥200 ng/dl	52	34(65.4%)	18(34.6%)	
Recurrence*				
Yes	34	23(67.6%)	11(32.4%)	0.454
No	86	64(74.4%)	22(25.6%)	
Tumor stage				
I	18	11(61.1%)	7(38.9%)	**<0.001**
II	32	14(43.8%)	18(56.2%)	
III	40	36(90%)	4(10%)	
IV	30	26(86.7%)	4(13.3%)	

Notes: Bold fonts indicate Statistically significant. Recurrence*:follow-up time for five years after therapeutic surgery.

**Table 2:** **COX regeression regression analysis on the relationship of clinicopathologic characteristics and prognosis**

Characteristics	Univariate			Multivariate		
	HR	CI(95%)	P	HR	CI(95%)	P
Mir-372	1.751	1.178-2.603	**0.006**	1.872	1.210-2.896	**0.005**
Age	0.982	0.671-1.437	0.924			
Gender	1.113	0.729-1.698	0.620			
Tumor stage	1.098	0.810-1.488	0.547			
Tumor differentiation	1.080	0.800-1.456	0.616			
Metastasis	1.942	1.319-2.861	**0.001**	1.811	1.131-2.898	**0.013**
Tumor size	1.334	0.896-1.986	0.156			
Serum AFP	2.280	1.482-3.505	**0.0001**	1.99	1.231-3.228	**0.005**
Cirrhosis	1.310	0.894-1.921	0.166			

Notes: Bold fonts indicate Statistically significant.

### Ectopic expression of miR-372 inhibits cancer cell line invasion

**Figure 7:**
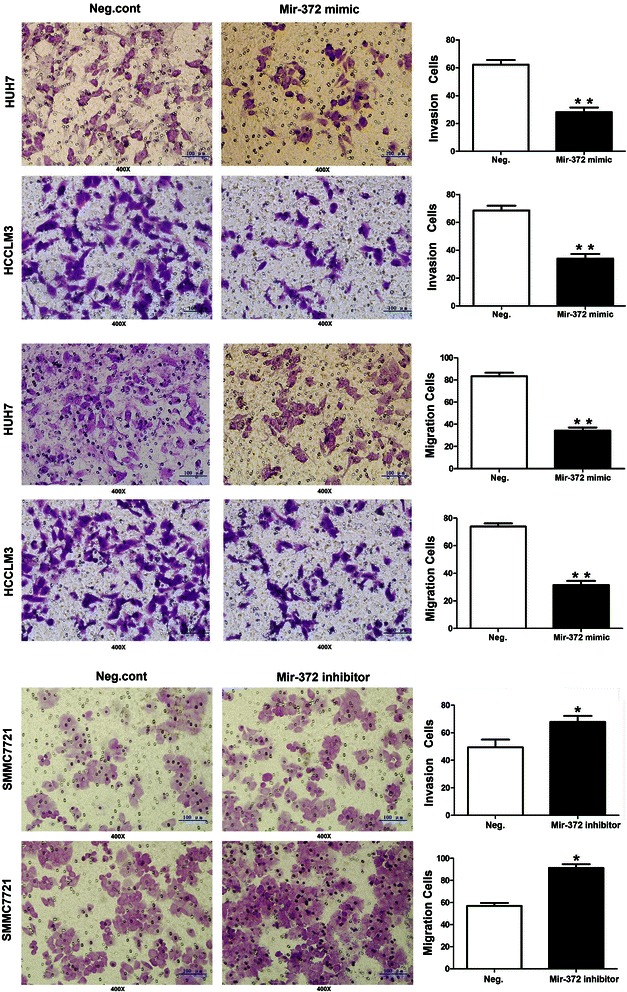
**Transwell assays of Huh7 and HCCLM3 cells transfected with neg.control and mir-372 mmic:mir-372 up-regulation had a measurable inhibitory effect on cell invasion in both cell lines.**

**Figure 8:**
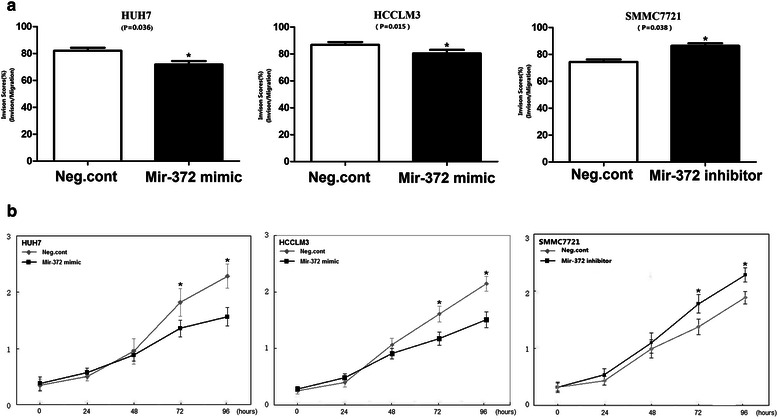
**Mir-372 expression levels could affect cellular invasion and proliferation in HUH7, HCCLM3 and SMMC7721 cells. a:** Invasion score was that the cell count in invasion assay divided by that of migration assay. **b:** The CCK8 assay was performed after mir-372 mimic and inhibitor treatment: A reduction of absorbance was observed in HUH7 and HCCLM3 (*P < 0.05); absorbance increased in SMMC7721 (*P < 0.05).

Of the seven liver cancer cell lines analyzed, Huh7 and HCCLM3 cells demonstrated relatively lower levels of miR-372 expression. Subsequently, these two cell lines were used to study the function of miR-372. We found that enhancement of the expression level of miR-372 in Huh7 and HCCLM3 cells could significantly inhibit invasion and migration abilities. Transwell chamber assay results showed that the number of invasive and migrated cells in the miRNA overexpression group (31 ± 6 and 39 ± 8, respectively) was significantly lower than that in the negative control group (62 ± 11 and 81 ± 14, respectively) in Huh7 cells. The same trend was also observed in HCCLM3 cells (30 ± 9 and 34 ± 7 vs. 67 ± 12 and 77 ± 16, respectively *P* < 0.01) (Figure 7). Because miR-372 could promote cell proliferation,so in order to eliminate strong effect on cell growth in Transwell assay we used the invasion score by calculating cell count in invasion assay divided by that of migration assay.Results showed the invasion score in miRNA overexpression group was significantly lower than that in the negative control group(71.8 ± 5.8% VS 82 ± 5.2% in Huh7 cells, *P* = 0.036;80.4 ± 5.8% VS 86.8 ± 4.6% in HCCLM3 cells, *P* = 0.015)(Figure 8a).

To evaluate the function of endogenous miR-372, we downregulated mir-372 expression in SMMC7721 cells (mir-372 high expression) by mir-372 inhibitor transfection, and found that cellular invasion and migration abilities were enhanced (invasion and migration of negative control group: 46 ± 6 and 59 ± 9, respectively, vs. mir-372 inhibitor group: 74 ± 13 and 86 ± 18, *P* < 0.05) (Figure 7).The invasion score in Neg.cont was lower than miR-372 inhibitor group (75.6 ± 4.3% VS 86.4 ± 3.9%, *P* =0.038)(Figure 8a).

### Ectopic expression of miR-372 inhibits cancer cell proliferation

**Figure 9:**
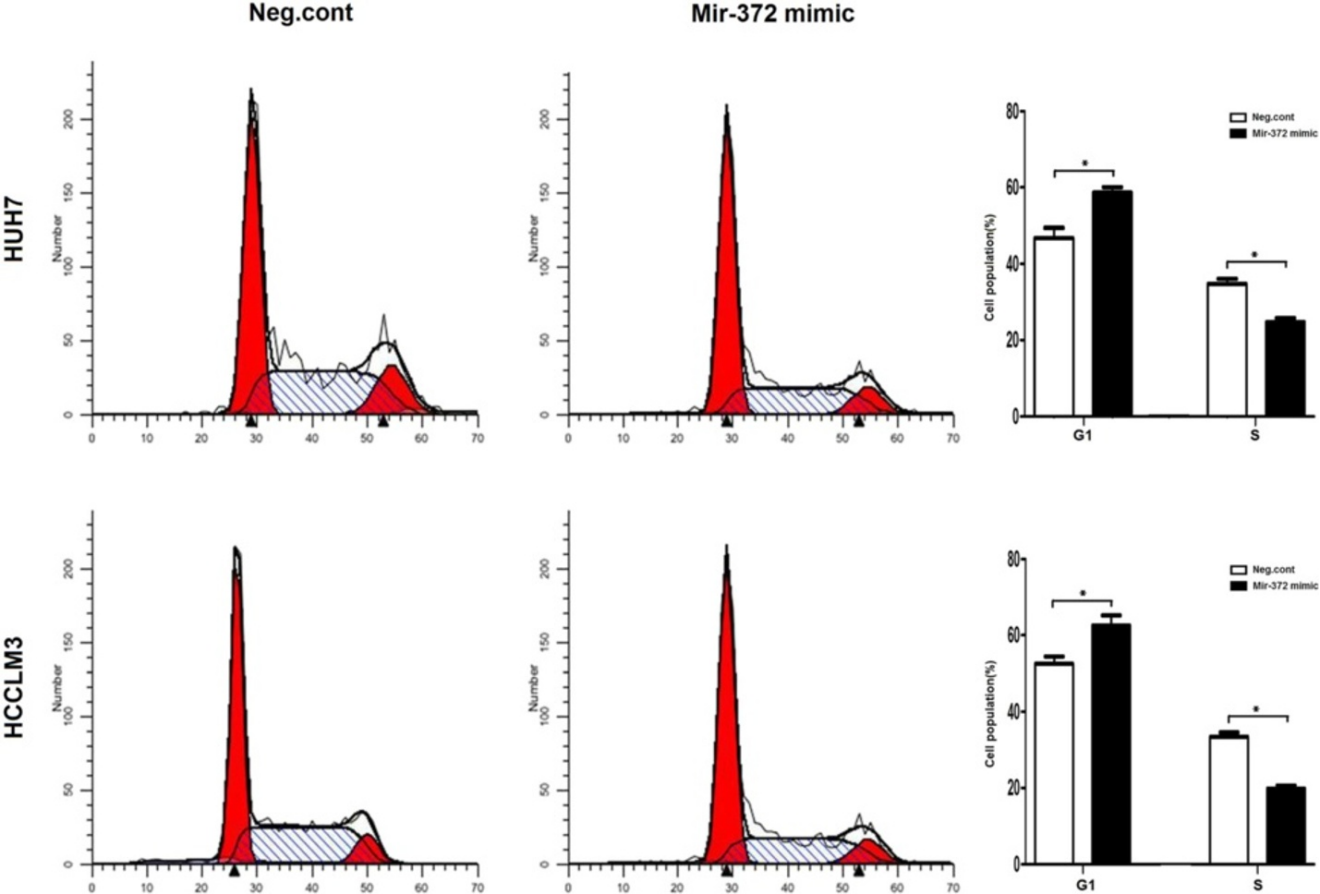
**Transfecting mir-372 mimic reduced Huh7 and HCCLM3 cell proliferation and led to a G1 phase cell cycle arrest.**

A significant reduction in the proliferation rate was observed by CCK8 assays 3 d after transfection with the miR-372 mimic compared with the negative control (Figure 8b). The effect of miR-372 on the cell cycle was tested using flow cytometry analysis. In Huh-7 cells, miR-372-overexpressed cells showed an increase in the number of cells in G1 phase (59.12%) and S phase (24.17%) compared with the negative control (G1, 45.63%; S, 36.76%). In HCCLM3 cells, miR-372-overexpression showed an increase in the number of cells in G1 phase (63.37%) and S phase (18.98%) compared with the negative control (G1, 52.14%; S, 33.59%) (Figure 9). Our results revealed that miR-372 could inhibit cell proliferation by blocking G1/S phase.

**Figure 10:**
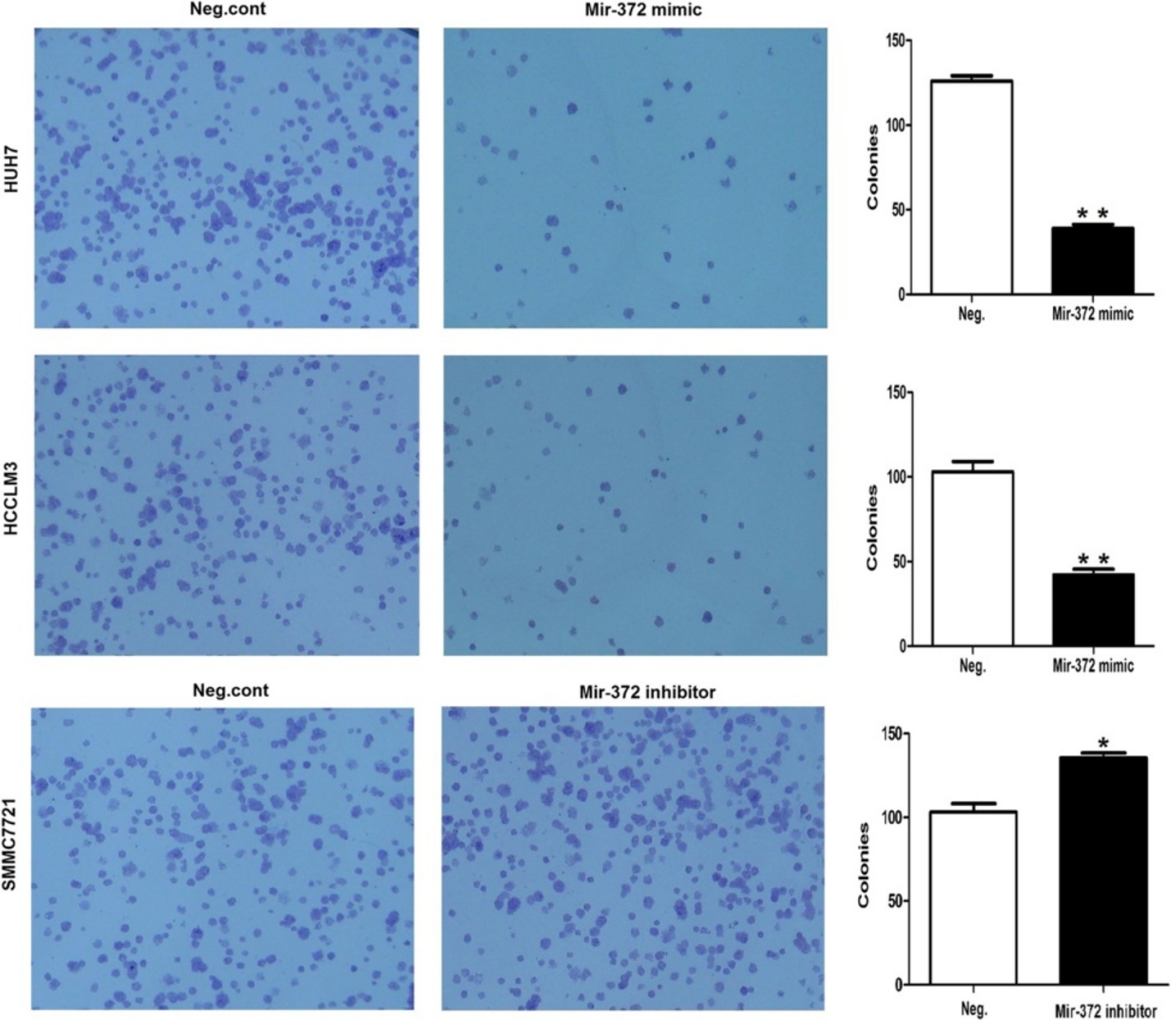
**Clonogenic assays were performed with HUH7/HCCLM3 cells (magnification × 10).** The number of colonies formed by cells treated with mir-372 mimic was far fewer than that of neg.cont-treated cells (P < 0.05).

Colony formation assay results in Huh-7 cells showed that the number of cells in the miR-372-overexpression group was significantly lower than that in the negative control group (123 ± 15 vs. 37 ± 9; *P* < 0.001). In addition, in HCCLM3 cells, the number of cells in the miR-372-overexpression group was significantly lower compared with the negative control group (104 ± 22 vs. 40 ± 11, *P* < 0.001) (Figure 10).

Moreover, downregulating mir-372 expression in SMMC7721 cells by miR-372 inhibitor transfection promoted proliferation as shown in CCK8 and colony formation assays (Figures 8b and 10). However, we did not find any alteration in cell cycle by flow cytometry analyses.

## Discussion

Many miRNAs have been shown to play important roles in regulating gene expression by mRNA cleavage or translational repression in a variety of model systems [17-19]. Profiling of miRNA expression showed that the majority of miRNAs are downregulated in tumors compared with normal tissues, such as miR-128 in glioma tissues [20] and miR-145 in human breast cancer [21]. Recent evidence suggests that miR-372 is a novel candidate tumorigenesis oncogene and possibly a therapeutic target in gastric cancer, non-small cell lung cancer, testicular germ cell tumors, and human glioma. However, it functions as an anti-tumor factor in cervical cancer [22]. Thus, miR-372 might serve different functions in different cellular environments by acting on different target genes.

The oncogene ATAD2 is a member of the AAA+ ATPase family of proteins that contains both a bromodomain and an ATPase domain, and the gene maps to chromosome 8q24 in a region that is frequently amplified in cancer [23]. ATAD2 promotes cellular proliferation and invasion by regulating the expression of downstream genes such as APC and CTNNA1 [14], both of which could play an important role in the development of HCC [24-26]. ATAD2 also targets PTCH1 [27], one of the key genes in the Hedgehog pathway, and regulates proliferation and differentiation in hepatic carcinogenesis. We previously identified miR-372 as a likely functional upstream target of ATAD2 in HCC using a luciferase reporter assay. In this study, we pursued the molecular function of miR-372 in liver cancer cells.

miR-372 belongs to the miR-371-373 gene cluster, which also includes miR-93 and miR-302a [28]. These miRNAs play an important role in the development of many types of human malignant tumors. However, reports have been conflicting regarding miR-372 expression levels in HCC, so in the current study we tested miR-372 expression not only by RT-PCR but also by *in situ* hybridization. Results from both approaches showed that miR-372 was expressed at low levels in HCC. We identified CpG islands upstream of the miR-372 promoter and speculated that aberrant promoter hypermethylation might be responsible for miR-372 low expression. The methylation status of neighboring CpG islands of mir-372 was tested by MSP both in HCC tissue samples and cell lines. MSP and RT-PCR showed the DNA methylation levels of miR-372 were significantly higher and the expression of miR-372 was significantly lower in tumors compared with their non-tumor tissue counterparts. Furthermore, DNA methylation status was related with the expression levels by Spearman rank analysis, suggesting that the expression of miR-372 was probably induced by aberrant DNA methylation. Moreover, in HCC cell lines with low expression of miR-372 (Huh7 and HCCLM3), treatment with 5-aza-dCyd could restore miR-372 expression but no changes were observed with TSA. Thus, this data suggests that aberrant promoter hypermethylation might induce the epigenetic silencing of miR-372 in HCC.

Transfection with miR-372 mimic in these two cell lines led to a G1 phase cell cycle arrest and reduced cell growth/proliferation. Similar results were also observed in CCK8 and colony formation assays. In addition, mimic-mediated miR-372 could significantly inhibit cell invasion in transwell chamber assays. Taken together, these data provide evidence that miR-372 is not only important in HCC cell proliferation but is also involved in cell invasion. Further analyses indicated that the low expression of miR-372 in the HCC tissues was negatively correlated with TNM stage and metastasis. These results demonstrated that the upregulation of miR-372 in HCC might play an important role in repressing malignant tumors. Similar results were also observed in cervical cancer, in which miR-372 was expressed at low levels and may downregulate CDK2 and cyclin A1 to control cell growth and cell cycle progression [22]. Furthermore, we found that the low expression of miR-372 in HCC was a strong and independent predictor of longer overall survival.

## Conclusions

In summary, here we clarified the anti-tumor role of miR-372 in HCC by four pieces of experimental evidence: (a) miR-372 is downregulated in HCC; (b) aberrantly high DNA methylation in the miR-372 gene promoter induced the epigenetic silencing of miR-372; (c) mir-372 can inhibit the proliferative and invasive capacity in HCC cell lines; and (d) miR-372 expression was related with tumor metastasis and poor prognosis in HCC.

These findings indicate that miR-372 plays an important role in hepatic carcinogenesis and is closely related to the outcome after HCC surgery. This may provide a new target or method to detect and treat HCC in the future. Therefore, further studies are still need to determine the precise mechanism underlying the role of miR-372 in HCC progression.
